# Factors Associated with Late Presentation of HIV and Estimation of Antiretroviral Treatment Need according to CD4 Lymphocyte Count in a Resource-Limited Setting: Data from an HIV Cohort Study in India

**DOI:** 10.1155/2012/293795

**Published:** 2012-04-24

**Authors:** Gerardo Alvarez-Uria, Manoranjan Midde, Raghavakalyan Pakam, Shanmugamari Kannan, Lakshminarayana Bachu, Praveen Kumar Naik

**Affiliations:** Department of Infectious Diseases, Rural Development Trust Hospital, Kadiri Road, Bathalapalli 515661, India

## Abstract

We describe the CD4 lymphocyte count at HIV presentation in an HIV cohort from a rural district of India. The majority of patients were diagnosed for their HIV-related symptoms, although a sizeable proportion of women were diagnosed because of antenatal screening or for having an HIV-positive partner. Patients diagnosed of HIV for antenatal screening or having an HIV-positive sexual partner had higher CD4 lymphocyte count than patients having tuberculosis or HIV-related symptoms. The proportion of patients diagnosed with CD4 count <200 and <350 cells/mm^3^ were 46% and 68.7%, respectively, and these figures did not change during the five years of the study. Factors associated with late presentations were male sex, older age, not having a permanent house, and, in women, lower education and being a widow or separated. With the implementation of 2010 WHO guidelines, the number of newly diagnosed patients who will require HIV treatment will increase 13.8%. If the CD4 count threshold for initiating HIV treatment is increased from 350 to 500 cells/mm^3^, the number of patients in need of treatment would increase 15.7%. Therefore, new strategies for avoiding HIV late presentation are urgently needed in developing countries.

## 1. Introduction

International HIV guidelines for the use of antiretroviral therapy (ART) in adults are shifting towards earlier initiation of HIV treatment [1–3]. Late initiation of ART is associated with higher risk of death and opportunistic infections as well as poorer response to treatment [4, 5]. However, patients who are diagnosed when their CD4 lymphocyte count is low are not able to get the benefits of an early ART initiation. From the public health point of view, these patients have a longer period of undiagnosed HIV infection. Late presentation of HIV delays patients from receiving education for avoiding the infection to others and precludes them from receiving ART, which can reduce their HIV viral load and, therefore, lowering the risk of HIV transmission to others.

In Europe and North America, approximately 30 to 35% of patients have CD4 lymphocyte count below 200 cells/mm^3^ at HIV diagnosis [6, 7]. Despite having more than 90% of the world burden of people living with HIV [8], information about the immunological situation at HIV diagnosis of patients from developing countries is scarce [9].

The aim of this study is to describe the CD4 lymphocyte count at HIV presentation in patients from a rural district of India and to investigate factors associated with late presentation [10]. We have also performed a simulation of how many people will require ART in this setting utilizing three different CD4 lymphocyte count thresholds for starting ART according to current guidelines [1–3, 11].

## 2. Patients and Methods

With 2.4 millions of HIV-infected people, India carries the largest population of people living with HIV in Asia and is the third country of the world in terms of HIV-infected people [12]. Andhra Pradesh is the state with highest burden of HIV in India, and HIV is transmitted mainly through heterosexual contacts [13]. Anantapur is a district with 74.7% of rural population situated in the south border of Andhra Pradesh [14]. Rural Development Trust (RDT) is a nongovernmental organization who has three hospitals in the district. In these hospitals, medical care of HIV-infected people is given free of cost, including medicines and consultation or admission charges. In Bathalapalli Hospital, the biggest hospital of RDT, outpatient clinics and 71 beds are allocated exclusively for HIV-infected patients where they can receive free specialized medical care for their opportunistic infections or other heath problems. Hence, most of people living with HIV in the district have visited our hospitals. We work in close collaboration with the ART Centres and Integrated Counselling and Testing Centres from the governmental Andhra Pradesh State AIDS Control Society.

The Vicente Ferrer HIV Cohort Study (VFHCS) is an open cohort study of all HIV-infected patients who have been visited in RDT hospitals since June 2006. Data from patients were collected prospectively since September 2009 and retrospectively from June 2006 to September 2009. Details of route of transmission, reason for HIV testing, and sociodemographic data are collected at enrolment. The VFHCS has been approved by the ethical committee of the RDT Institutional Review Board.

All patients from the VFHCS living in the district of Anantapur, aged above 15 years, who were diagnosed of HIV between June 2006 and March 2011 and who had at least one determination of CD4 lymphocyte count before starting ART were included in the study. Measurement of CD4 lymphocyte count was performed using flow cytometry (Cyflow SL, Partec, Munster, Germany). HIV diagnosis was performed following the guidelines of the National AIDS Control Organization of India [15], and counselling was given before performing the HIV test and after knowing the result of the test.

Patients whose first CD4 lymphocyte count was below 350 cells/mm^3^ were defined as late presenters following current recommendations [10]. Logistic regression was used for the multivariable analysis of factors associated with late presentation of HIV infection using Stata Statistical Software (Stata Corporation, Release 11, College Station, TX, USA) [16]. Missing values were imputed using multiple imputations by chained equation [17]. Confidence intervals (CIs) for the difference in the proportion of patients who will require HIV treatment using three different CD4 count thresholds were calculated using the Agresti-Caffo procedure. This method is considered to have better performance than the traditional Wald method for calculating the CIs for the difference between two independent proportions [18]. Because CD4 lymphocyte determinations are performed approximately every six months, life graphs for the proportion of patients who would require initiation of antiretroviral treatment according to three different CD4 lymphocyte count thresholds were calculated at 6-month intervals.

## 3. Results

**Figure 1:**
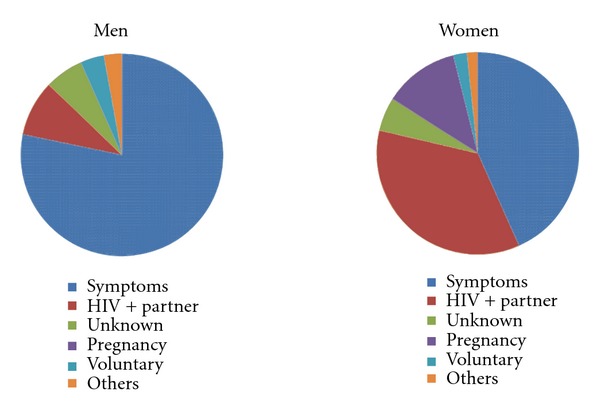
Reasons for HIV diagnosis in women and men.

**Figure 2:**
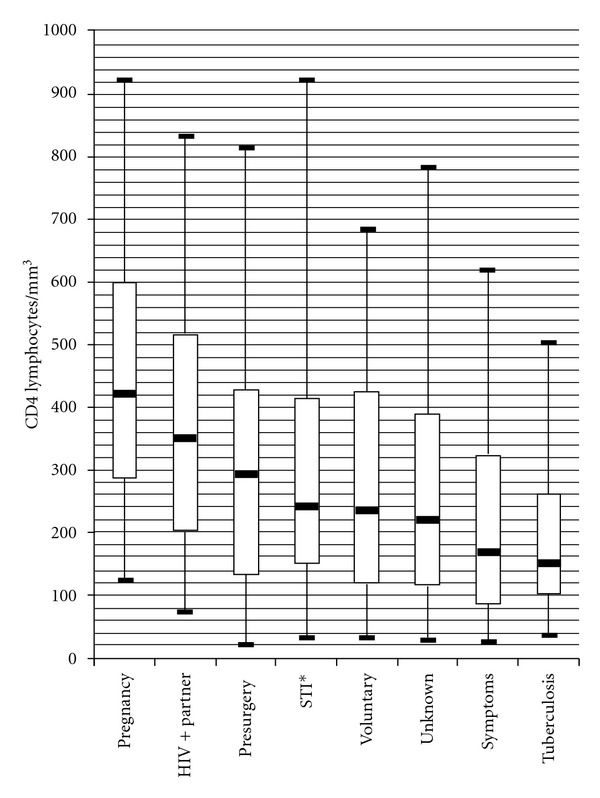
Description of CD4 lymphocyte count by reason for HIV testing in descending order from right to left. Boxes represent median and 25–75 percentiles. Whiskers represent 5 and 95 percentiles. *STI: sexually transmitted infection.

Out of 7905 HIV-positive patients diagnosed of HIV during the period of the study, 6215 (78.6%) had at least one CD4 lymphocyte count determination before starting ART and were included in the study. Reasons for HIV testing are presented in Table 1. Most of the patients were diagnosed for having HIV-related symptoms. Differences in the reasons for HIV diagnosis between men and women are shown in Figure 1. In women, the proportion of patients diagnosed for having an HIV-positive partner was higher than in men (*P* < 0.001). Median and interquartile range (IQR) of CD4 lymphocyte count at HIV presentation was 218 (IQR, 108–402). Median CD4 lymphocyte count at presentation was 287 (IQR, 138–466) for women and 180 (IQR, 91–331) for men (*P* < 0.001). Relation between CD4 lymphocyte count and reason for HIV testing are presented in Figure 2. We did not observe gender differences in CD4 lymphocyte counts when stratifying by reason for HIV testing.

**Figure 3:**
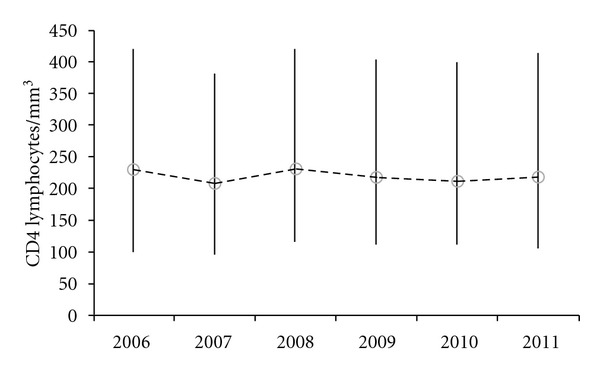
Median and interquartile range of CD4 lymphocyte count at HIV presentation by calendar year of presentation.

Median and IQR of CD4 lymphocyte count at presentation in each calendar year during the period of the study are shown in Figure 3. The figure shows that the CD4 lymphocyte count at HIV presentation has remained stable during the five years of the study.

Multivariable analysis of factors associated with late presentation is described in Table 2. Factors associated with late HIV presentation were older age and not having a permanent house. No difference was seen among communities, monthly income, living near a city, route of transmission, or consuming alcohol. In women, late presentation was more common among those with lower education or who were widow or separated. Among men, weavers were less likely to have late presentation.

**Figure 4:**
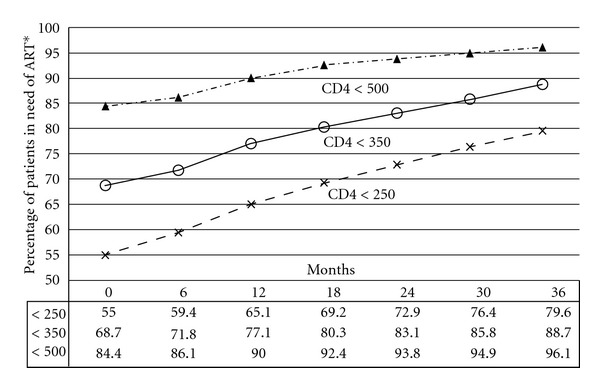
Cumulative incidence of patients who will require antiretroviral treatment according to three different thresholds for initiating HIV treatment after 0, 6, 12, 18, 24, 30 and 36 months since the first determination of CD4 lymphocyte count. *ART: antiretroviral therapy.

Proportions of patients for four CD4 lymphocyte count thresholds at presentation are presented in Table 3. Approximately, two-thirds of patients presented with a CD4 lymphocyte count below 350 cells/mm^3^ and 46% with CD4 lymphocyte count below 200 cells/mm^3^.  Figure 4 represents the cumulative incidence of newly diagnosed patients who will require ART according to three different thresholds for starting HIV treatment after 0, 6, 12, 18, 24, 30, and 36 months since the first determination of CD4 lymphocyte count. For constructing this graph, all CD4 lymphocyte count determinations before starting ART were taken into account. Current National AIDS Control Organization of India guidelines for HIV treatment recommend starting ART when the CD4 lymphocyte count is below 250 cells/mm^3^ following previous WHO recommendation for initiating ART before patients had CD4 lymphocyte count below 200 cells/mm^3^ [11]. With the 2010 World Health Organization (WHO) guidelines, the threshold for starting HIV treatment has been increased to 350 CD4 cells/mm^3^ [3]. According to our data, the implementation of 2010 WHO guidelines will increase the number of patients who will need antiretroviral treatment after the first determination of CD4 lymphocyte count in 13.8% (95% CI 12.1–15.5). If the threshold is increased from 350 to 500 CD4 cells as is contemplated in other guidelines [1, 2], the number of patients who will require treatment would increase 15.7% (95% CI 14.2–17.1). These differences are progressively reduced over time.

## 4. Discussion

In this study we found that an important proportion of HIV-infected patients presented with low CD4 lymphocyte count. In another study also performed in India in an urban reference hospital of New Delhi, the proportion of patients who presented with CD4 cell count below 200 cells/mm^3^ was lower than in our study [19]. This may reflect a later presentation of HIV in rural settings compared to urban settings in India. When comparing our results with other study performed in Nigeria, CD4 count at presentation was lower in African women, but differences were not important between Nigerian and Indian men [20]. Considering the three studies, the proportion of patients who present with CD4 lymphocyte count below 200 cells/mm^3^ in developing countries can be probably between 40 and 50%. More studies addressing late presentations of HIV in the developing world are urgently needed. We must remember that effective ART is one of the most effective measures to reduce the transmission of HIV [21]. Increasing the threshold of CD4 lymphocyte count for starting ART is important, but it should be accompanied by the implementation of new strategies for achieving earlier diagnosis of HIV. As seen in Figure 3, the CD4 lymphocyte count at presentation remained stable during the period of the study, indicating that if we do not change the way HIV is diagnosed, we will continue to face the problem of late presentation of HIV in India.

The results of this study show the relation between the CD4 lymphocyte count at presentation and the reason for HIV testing. This observation is essential for designing public health interventions orientated to achieve early detection of HIV infection. In the majority of patients, the HIV diagnosis was made because a health worker suspected that their symptoms could be explained by an HIV infection, leading to a late diagnosis of HIV. When the reason for testing was related to having risk factors for HIV in asymptomatic patients, like having sexually transmitted diseases or an HIV-positive partner, CD4 lymphocyte count at presentation was higher than patients with tuberculosis or HIV-related symptoms (*P* < 0.001). The best results in terms of early diagnosis were seen in the screening of pregnant women. However, strategies that involve massive HIV testing to the population are not feasible in a highly populated country with relatively low prevalence of HIV such as India. Interventions that involve facilitating HIV testing in all types of health facilities, including small and rural clinics, for patients who have risk factors for HIV infection can be a better cost-effective option. It is important to generalize the use of the HIV testing in India. Integrated Counselling and Testing Centres are able to provide good pre- and posttest counselling, but patients are reluctant to attend them due to the problem of HIV-associated stigma in India.

We found important differences in factors associated with late HIV presentation between women and men. In women, late presentation was associated with lower education level and being widow, separated, or divorced indicating the difficulties of reaching the health care system for women in the Indian rural setting. In an urban tertiary hospital care setting [19], late presentation was seen to be associated with male sex and older age.

With the implementation of 2010 WHO ART guidelines in India, more than two-thirds of newly diagnosed HIV-infected patients will require immediate initiation of ART. This can be challenging because side effects of ART are common and many of these patients will be asymptomatic prior to ART initiation. It is essential to provide intensive counselling about the possible side effects of the drug and the long-term benefits of the medication to avoid patients to be lost during followup. In terms of cost of antiretrovirals and not taking into account the economical benefits of an earlier initiation of ART [22, 23], the number of patients who will require ART will increase by around 14%. However, if we observe Figure 4, this difference decreases over the time reaching approximately 9% after three years. Also looking at Figure 4, increasing the threshold for ART initiation from 250 to 350 cells/mm^3^ and from 350 to 500 cells/mm^3^, we are actually bringing forward approximately 18 and 27 months, respectively, the initiation of ART in the population.

The study has some drawbacks. When estimating the antiretroviral need of the population, we have not taken into consideration clinical data from the patients. Some of the patients with CD4 lymphocyte count between 250 and 350 cells/mm^3^ may have required initiation of ART because of their HIV-related symptoms. Also, information about the reason for HIV testing and route of transmission was performed through direct questioning to the patients. Patients may have been reluctant to answer when interrogated about sexually transmitted diseases or homosexual contacts leading to an underrepresentation of these factors in the study. In addition, data from patients who did not attend our clinics after September 2009 were collected retrospectively using the clinical notes of the patients, so some of these data were missed if no information was present in the clinical notes.

## 5. Conclusions

The results of this study can have important implication for designing public health intervention in developing countries. The proportion of HIV late presenters in this Indian rural setting, which has remained stable in the last five years, was higher than the proportion of late presenters reported in western countries. Most of the patients are diagnosed of HIV when they experience HIV-related symptoms, especially men. New strategies for reducing the late presentation of HIV should focus on screening of patients with risk factors for having HIV infection when they are still asymptomatic. With the implementation of 2010 WHO guidelines for ART, two-thirds of HIV patients will require immediate initiation of ART after the first determination of CD4 lymphocyte count.

## Figures and Tables

**Table 1:** Reasons for HIV diagnosis.

Reason for HIV testing	*N*	%
HIV-related symptoms	3857	62.06
HIV+ partner	1314	21.14
Pregnancy screening	348	5.60
Voluntary	189	3.04
Presurgery	54	0.87
Tuberculosis	53	0.85
Sexually transmitted infection	31	0.50
Blood donation	6	0.10
Vertical transmission	4	0.06
Unknown	359	5.78

Total	6215	100

**Table 2:** Factors associated with late HIV presentation stratified by gender.

	Women				Men			
	CD4 count	CD4 count <350/mm^3^			CD4 count	CD4 count <350/mm^3^		
	Median (IQR)	Proportion (%)	aOR	95% CI	Median (IQR)	Proportion (%)	aOR	95% CI
Age (years)								
15 to 24	373 (223–548)	464/999 (46.4)	0.58***	(0.48–0.71)	249 (128–414)	226/336 (67.3)	0.71*	(0.54–0.94)
25 to 34	271 (133–447)	744/1213 (61.3)	1	Reference	185 (94–353)	1122/1512 (74.2)	1	Reference
35 to 44	190 (95–355)	348/473 (73.6)	1.64***	(1.26–2.12)	159 (79–287)	804/999 (80.5)	1.47***	(1.20–1.80)
≥45	160 (84–293)	156/191 (81.7)	2.48***	(1.62–3.79)	161 (87–302)	406/492 (82.5)	1.67***	(1.26–2.20)

Marital status								
Single	217 (105–429)	18/27 (66.7)	1.73	(0.62–4.78)	197 (105–353)	241/328 (73.5)	1.09	(0.81–1.48)
Married	315 (162–510)	941/1705 (55.2)	1	Reference	180 (91–332)	1987/2597 (76.5)	1	Reference
Separated-divorced	225 (111–435)	134/200 (67)	1.44*	(1.01–2.05)	139 (82–293)	110/134 (82.1)	1.41	(0.88–2.25)
Widow	241 (118–408)	581/881 (65.9)	1.24*	(1.01–1.53)	156 (80–280)	161/202 (79.7)	1	(0.69–1.46)

Education								
Higher	355 (166–525)	25/54 (46.3)	0.50*	(0.26–0.96)	212 (112–368)	119/167 (71.3)	0.86	(0.57–1.29)
Secondary	367 (197–546)	264/567 (46.6)	0.58***	(0.46–0.73)	189 (92–343)	804/1065 (75.5)	1.02	(0.83–1.26)
Primary	325 (170–532)	166/307 (54.1)	0.79	(0.59–1.04)	171 (94–315)	504/638 (79)	1.14	(0.90–1.45)
No education	253 (120–428)	1219/1884 (64.7)	1	Reference	168 (88–325)	1077/1397 (77.1)	1	Reference

Community								
Other castes	294 (134–477)	378/641 (59)	0.93	(0.75–1.16)	175 (87–340)	674/887 (76)	0.93	(0.76–1.14)
Backward castes	290 (140–461)	842/1414 (59.5)	1	Reference	184 (95–330)	1203/1573 (76.5)	1	Reference
Scheduled castes	266 (126–452)	373/603 (61.9)	1.06	(0.85–1.33)	174 (85–313)	532/672 (79.2)	1.14	(0.90–1.43)
Scheduled tribes	303 (168–498)	119/218 (54.6)	0.77	(0.56–1.07)	175 (104–379)	149/207 (72)	0.77	(0.55–1.09)

House type								
Owned	295 (150–470)	773/1321 (58.5)	1	Reference	185 (101–340)	1166/1536 (75.9)	1	Reference
Rented	314 (167–473)	513/925 (55.5)	0.91	(0.75–1.10)	197 (101–361)	673/910 (74)	0.98	(0.80–1.20)
No house	200 (90–402)	202/292 (69.2)	1.50*	(1.10–2.04)	134 (67–267)	323/383 (84.3)	1.60**	(1.18–2.17)
Other	274 (118–454)	59/97 (60.8)	1.05	(0.67–1.66)	135 (80–294)	159/201 (79.1)	1.39	(0.96–2.02)

Monthly income (INR^a^)								
<1000	281 (140–460)	715/1204 (59.4)	1	Reference	178 (90–325)	942/1223 (77)	1	Reference
1001 to 2000	287 (154–432)	204/339 (60.2)	0.98	(0.74–1.30)	182 (97–325)	547/705 (77.6)	1.12	(0.89–1.42)
2001 to 3000	270 (119–443)	71/111 (64)	1.25	(0.79–1.96)	182 (92–369)	236/323 (73.1)	0.92	(0.69–1.24)
>3000	303 (148–458)	76/135 (56.3)	1	(0.65–1.54)	183 (89–353)	529/707 (74.8)	0.93	(0.74–1.19)
Not applicable	297 (144–493)	489/839 (58.3)	1.13	(0.86–1.48)	160 (89–299)	224/280 (80)	1.18	(0.82–1.69)

Occupation								
Unskilled labourer	257 (122–435)	932/1472 (63.3)	1	Reference	170 (87–323)	947/1219 (77.7)	1	Reference
Driver					180 (93–330)	307/406 (75.6)	1.05	(0.79–1.40)
Farmer	379 (211–536)	40/84 (47.6)	0.73	(0.44–1.21)	161 (86–304)	362/454 (79.7)	1.19	(0.89–1.59)
Health worker	304 (177–551)	22/41 (53.7)	1.11	(0.54–2.30)	250 (127–500)	5/10 (50)	0.4	(0.11–1.49)
Housekeeper	336 (176–521)	398/752 (52.9)	0.91	(0.69–1.21)	388 (220–594)	2/5 (40)	0.18	(0.03–1.18)
Weaver	319 (226–488)	16/30 (53.3)	0.91	(0.40–2.09)	272 (118–426)	42/70 (60)	0.52*	(0.31–0.87)
Others	273 (140–463)	220/367 (59.9)	1.11	(0.85–1.45)	193 (98–343)	789/1049 (75.2)	1	(0.80–1.24)
Unemployed	168 (100–356)	40/57 (70.2)	0.99	(0.50–1.98)	131 (82–261)	49/56 (87.5)	2.13	(0.89–5.12)

Living near a city^b^								
No	286 (136–458)	966/1610 (60)	1	Reference	171 (87–318)	1466/1874 (78.2)	1	Reference
Yes	286 (138–470)	746/1266 (58.9)	1	(0.84–1.19)	189 (97–353)	1092/1465 (74.5)	0.87	(0.73–1.03)

Route of transmission								
Heterosexual	291 (140–468)	1443/2453 (58.8)	1	Reference	178 (90–326)	2446/3174 (77.1)	1	Reference
Blood transfusion	263 (129–479)	43/66 (65.2)	1.15	(0.61–2.16)	191 (92–315)	17/22 (77.3)	0.93	(0.33–2.60)
Homosexual					386 (313–406)	2/5 (40)	0.36	(0.05–2.47)
Others	250 (139–363)	14/21 (66.7)	1.48	(0.42–5.23)	214 (102–387)	17/24 (70.8)	0.7	(0.27–1.83)
Alcohol consumption								
Yes	282 (153–431)	15/20 (75)	1.58	(0.41–6.07)	173 (89–324)	1592/2064 (77.1)	0.99	(0.83–1.19)
Never	288 (140–462)	1579/2667 (59.2)	1	Reference	192 (95–345)	911/1200 (75.9)	1	Reference

^a^Indian Rupees (approximately 1$ = 46 INR in September 2011).

^b^population >100,000 people.

***P < 0.001, **P < 0.01, *P < 0.05.

**Table 3:** Proportions and Wilson 95% confidence intervals for four different CD4 cell count thresholds at HIV presentation.

	Total (*n* = 6215)		Women (*n* = 2876)		Men (*n* = 3339)	
CD4 cells/mm^3^	Proportion	95% CI	Proportion	95% CI	Proportion	95% CI
<50	8.9%	(8.2–9.7)	6.3%	(5.5–7.3)	11.2%	(10.1–12.3)
<200	46.0%	(44.7–47.2)	35.7%	(34.0–37.5)	54.8%	(53.1–56.5)
<350	68.7%	(67.5–69.8)	59.5%	(57.7–61.3)	76.6%	(75.1–78.0)
<500	84.4%	(83.4–85.2)	78.4%	(76.9–79.9)	89.5%	(88.4–90.5)
